# Short Telomere Length Is Related to Limitations in Physical Function in Elderly European Adults

**DOI:** 10.3389/fphys.2018.01110

**Published:** 2018-08-10

**Authors:** Diego Montiel Rojas, Andreas Nilsson, Elodie Ponsot, Robert J. Brummer, Susan Fairweather-Tait, Amy Jennings, Lisette C. P. G. M. de Groot, Agnes Berendsen, Barbara Pietruszka, Dawid Madej, Elodie Caumon, Nathalie Meunier, Corinne Malpuech-Brugère, Giulia Guidarelli, Aurelia Santoro, Claudio Franceschi, Fawzi Kadi

**Affiliations:** ^1^School of Health Sciences, Örebro University, Örebro, Sweden; ^2^Norwich Medical School, University of East Anglia, Norwich, United Kingdom; ^3^Department of Human Nutrition, Wageningen University & Research, Wageningen, Netherlands; ^4^Department of Human Nutrition, Warsaw University of Life Sciences, Warsaw, Poland; ^5^Centre Hospitalier Universitaire de Clermont-Ferrand, Centre de Recherche en Nutrition Humaine d’Auvergne, Clermont-Ferrand, France; ^6^Unité de Nutrition Humaine, Institut National de la Recherche Agronomique, Centre de Recherche en Nutrition Humaine d’Auvergne, Université Clermont Auvergne, Clermont-Ferrand, France; ^7^Department of Experimental, Diagnostic and Specialty Medicine, University of Bologna, Bologna, Italy; ^8^Interdepartmental Center “L. Galvani” (CIG), University of Bologna, Bologna, Italy; ^9^Bellaria Hospital, Institute of Neurological Sciences, University of Bologna, Bologna, Italy

**Keywords:** aging, ethnicity, handgrip strength, SF-36, physical function

## Abstract

The present study aims to explore the potential influence of leucocyte telomere length (LTL) on both a single indicator and a composite construct of physical functioning in a large European population of elderly men and women across diverse geographical locations. A total of 1,221 adults (65–79 years) were recruited from five European countries within the framework of NU-AGE study. The physical functioning construct was based on the 36-item Short Form Health Survey. Handgrip strength was used as a single indicator of muscle function and LTL was assessed using quantitative real-time PCR. Women had significantly longer (p < 0.05) LTL than men. Participants in Poland had significantly shorter LTL than in the other study centers, whereas participants in the Netherlands had significantly longer LTL than most of the other centers (p < 0.01). An analysis of LTL as a continuous outcome against physical functioning by using linear models revealed inconsistent findings. In contrast, based on an analysis of contrasting telomere lengths (first vs. fifth quintile of LTL), a significant odds ratio (OR) of 1.7 (95% CI: 1.1 – 2.6; p < 0.05) of having functional limitation was observed in those belonging to the first LTL quintile compared to the fifth. Interestingly, having the shortest LTL was still related to a higher likelihood of having physical limitation when compared to all remaining quintiles (OR: 1.5, 95% CI: 1.1 – 2.1; p < 0.05), even after adjustment by study center, age, sex, and overweight status. Collectively, our findings suggest that short LTL is an independent risk factor that accounts for functional decline in elderly European populations. The influence of LTL on functional limitation seems driven by the detrimental effect of having short telomeres rather than reflecting a linear dose-response relationship.

## Introduction

Increased life expectancy is accompanied by a progressive increase in physical functioning limitations, defined as the difficulty in performing basic daily activities (Fried, 2016). Although several environmental and lifestyle factors may contribute to functional deterioration, the role of biological mechanisms underlying this age-related decline are largely unknown.

Telomeres are repetitive nucleotide sequences located at the terminal ends of chromosomes, contributing to genomic integrity and stability (Stewart et al., 2012). Telomere shortening occurs at each round of cell division and is influenced by oxidative stress (Harley et al., 1990; Von Zglinicki, 2002). When telomere length drops below a critical threshold, the proliferative capacity of tissues becomes impaired. In fact, short telomeres have been linked to higher risk of mortality in elderly adults (Cawthon et al., 2003; Weischer et al., 2012) and accelerated telomere shortening has been reported in patients with cardiovascular and metabolic disorders (Oeseburg et al., 2010; Xi et al., 2013; Marzetti et al., 2014).

Previous investigations of links between leucocyte telomere length (LTL) and physical functioning during old age have yielded inconclusive results (Harris et al., 2006, 2012, 2016; Risques et al., 2010; Bendix et al., 2011; Lee et al., 2013; Woo et al., 2014; Sillanpää et al., 2016), where between-study inconsistencies may be related to the use of different approaches in assessing physical functioning. Notably, some of the previous studies have used single tests of muscle function whereas others have used constructs based on self-reported abilities in the assessment of physical functioning (Harris et al., 2006, 2012, 2016; Risques et al., 2010; Bendix et al., 2011; Lee et al., 2013; Woo et al., 2014; Sillanpää et al., 2016). It can be hypothesized that in contrast to a composite construct of self-reported abilities, single tests of muscle function may not fully capture the inability to perform daily activities. More importantly, consistent differences in LTL have been indicated in geographically diverse populations (Hunt et al., 2008), where a 25% difference in LTL was demonstrated in young adults across European countries (Eisenberg et al., 2011). Therefore, it is critical to consider these differences when investigating links between LTL and health outcomes across diverse geographical locations.

In order to determine the importance of biological factors underlying functional decline in aging populations, we aimed to explore the potential influence of LTL on both a single indicator and a construct of physical functioning in a large sample of 1,221 elderly men and women from the NU-AGE project cohort, involving five European countries.

## Materials and Methods

### Study Population

Data from 1,221 older adults (536 men and 685 women, aged 65–79 years) recruited within the NU-AGE project^1^ (registered with clinicaltrials.gov, NCT01754012), a multi-center study exploring determinants of healthy aging across five European countries (France, Italy, Netherlands, Poland, and the United Kingdom), were used in this study. A detailed description of the study population can be found elsewhere (Berendsen et al., 2014; Santoro et al., 2014). Subjects with overt issues such as organ failure, severe heart disease, chronic kidney disease, respiratory insufficiency, liver cirrhosis, diabetes mellitus type I, malnutrition, or who were unable to give informed consent were excluded. Written informed consent was obtained from participants and the study conducted in accordance with standards set by the Declaration of Helsinki. Ethical approval was obtained from local ethical review boards at each participating study center.

### Assessment of Relative Leucocyte Telomere Length

Total genomic DNA was purified from peripheral whole blood using a commercially available kit (Nucleospin Blood DNA, Macherey–Nagel) according to the manufacturer’s instructions and stored at -20°C for subsequent use. LTL was assessed using an established quantitative real-time PCR technique (Cawthon, 2002). Real-time PCR for telomere (T) and single copy gene expression (S) was performed using Rotor-Gene SYBR Green RT-PCR Master Mix (Qiagen) on Rotor-Gene Q (Qiagen) according to the manufacturer’s instructions. Oligomer sequences for telomere were 5′ CGG TTT GTT TGG GTT TGG GTT TGG GTT TGG GTT TGG GTT 3′ (forward) and 5′ GGC TTG CCT TAC CCT TAC CCT TAC CCT TAC CCT TAC CCT 3′ (reverse). The sequences for 36B4 were 5′ CAG CAA GTG GGA AGG TGT AAT CC 3′ (forward) and 5′ CCC ATT CTA TCA TCA ACG GGT ACA A 3′ (reverse) (Brouilette et al., 2007). Samples were loaded in duplicates for 36B4 and in triplicates for telomere. The specificity of PCR products amplification was confirmed by melting curves, and amplification efficiencies were validated by standard dilutions series. An internal control was loaded in each run. Telomere length was expressed as T/S ratio using the 2^-ΔΔCT^ method with a CV of 4.6 ± 0.4% (mean ± SE).

### Physical Functioning Limitation

Physical Functioning (PF) was assessed by the 10-item PF subscale of the 36-item Short Form Health Survey (SF-36) (Syddall et al., 2009); a tool widely evaluated in term of construct validity and reliability in diverse ethnic groups of different ages. The PF subscale uses a 3-item response scale (limited a lot; limited a little; not limited at all) to address 10 questions related to the ability to accomplish various daily activities. Subjects who reported “limited a lot” on at least one of the 10 questions were classified as having physical limitation, and all other participants were subsequently classified as not having any physical limitation. Handgrip strength, a single indicator of muscle function, was additionally assessed by standardized procedures using Jamar handheld dynamometer (Patterson Medical, Warrenville, IL, United States).

### Statistical Analysis

Due to skewness, LTL was transformed (log10) to fulfill assumptions behind parametric analyses. Differences in LTL between sexes and study centers were performed with analysis of variance (ANOVA), adjusted for multiple comparisons by Sidak correction.

Links between LTL and discrete constructs of physical limitations were analyzed using two approaches; one based on LTL as a continuous variable in parametric modeling of the whole sample, and the other based on the group of participants who had the shortest LTL compared to those with longer LTL based on non-parametric modeling.

First, general linear models were employed, where the constructs of having physical limitation, based either on self-report (yes/no) or handgrip strength (in quintiles and normalized by body weight), were set as fixed-factors with LTL as dependent. As both outcome and predictors are gender-sensitive, analyses were stratified by sex. Prior to the main analysis, the potential influence of covariates on LTL was tested within each stratified model, with *p* ≥ 0.1 set as F-to-remove criteria. As a result, study center was included in both stratified models, whereas age and overweight status (BMI ≥ 25 kg/m^2^) were included only when modeling data specifically in women. Subsequently, medication use, smoking status, and alcohol intake were excluded.

Second, quintiles of LTL were derived within groups of men and women, identifying those men and women who had the shortest LTL (q1), compared with those who had the longest (q5), as well as all other groups combined (q2 + q3 + q4 + q5). As the LTL quintile groups were derived from within men and women, subsequent analyses were conducted without stratification. The proportion differences of having functional limitation (self-reported: yes/no; handgrip: q1/q5) between LTL subgroups were analyzed with logistic regression. Here, the likelihood of having physical limitation was compared between those who had the shortest LTL compared to those with longer LTL, accounting for sex, age, study center, and overweight status (BMI ≥ 25 kg/m^2^), which all influence on the likelihood of having physical limitation. The level of statistical significance was set to *p* < 0.05. All statistical procedures were performed using SPSS version 24.

## Results

Based on the composite construct of physical functioning, 30.5% of the elderly adults reported functional limitation, with a significantly (*p* < 0.05) higher proportion of women compared to men (**Table 1**). As expected, women had significantly (*p* < 0.05) lower handgrip strength compared to men, and a total of 65% (70% men and 61% women) of the study sample was classified as overweight (**Table 1**).

**Table 1:** Characteristics of the study sample by gender.

	Total	Male	Female
	***n* = 1221**	***n* = 536**	***n* = 685**
Age, years	70.9 ± 4.0	71.1 ± 4.1	70.7 ± 3.9
LTL, T/S Ratio	0.85 ± 0.25	0.83 ± 0.24	0.87 ± 0.25^∗^
Handgrip strength, kg	31.5 ± 9.5	39.6 ± 6.9	25.2 ± 5.5^∗^
Having physical limitation^a^, %	30.5	20.3	38.5^∗^
Overweight^b^, %	64.8	69.8	60.9^∗^
Former-current smokers, % yes	46.8	59.3	37.1^∗^
Medication, % yes	77.5	76.9	78.0


*LTL, Leucocyte Telomere Length. ^*a*^Based on composite construct. ^*b*^Based on BMI ≥ 25 kg/m^2^. Data expressed as mean ±*SD*, unless indicated. ^∗^*p* < 0.05 vs.male.*

**FIGURE 1:**
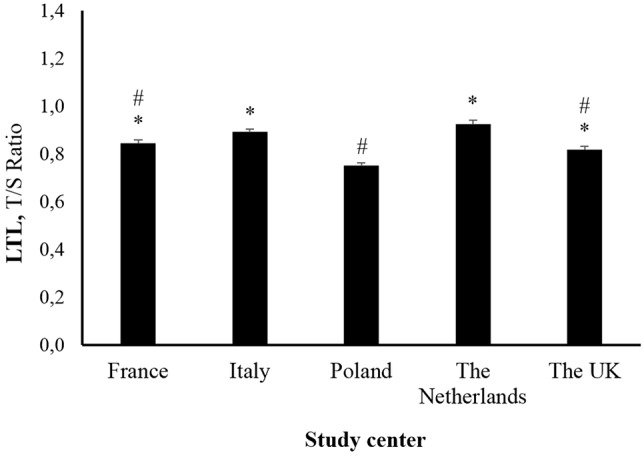
Leucocyte telomere length in older adults across study centers. Data are estimated marginal means ± SE. ^∗^*p* < 0.05 vs. Poland. #*p* < 0.05 vs. Netherlands.

Leucocyte telomere length evaluation in our study sample revealed the influences of sex and study center, with women having significantly longer (*p* < 0.05) LTL than men. Between study centers, participants in Poland had significantly shorter LTL than all other study centers, whereas participants in the Netherlands presented significantly longer LTL than most of the other centers (*p* < 0.01). Importantly, between-study center differences in LTL remained after adjustment for sex and age (**Figure 1**).

Based on general linear models using LTL as a continuous variable, functionally limited elderly adults had shorter LTL compared to their non-limited peers in both sexes. After further adjustment by study center, the significant effect remained in men (*p* < 0.05), but was attenuated in women. In contrast to findings observed using the composite construct, no differences in LTL across quintiles of handgrip strength were observed for males or females.

Further, based on quintiles of LTL, we sought to investigate the influence of having short telomeres on physical functioning. First, we selected a sub-sample comprising the first and fifth quintiles (the shortest vs. the longest), where about one third (32%) had functional limitations (**Table 2**). Among those with the shortest LTL, 24% and 50% were physically limited in men and women, respectively. Logistic regression analysis showed a significantly higher proportion having functional limitation among those with the shortest LTL compared to those with the longest regardless of the sex. After further adjustment by age, overweight status, and study center, a significant odds ratio (OR) of 1.7 (95% CI: 1.1 – 2.6; *p* < 0.05) of having functional limitation depending on LTL grouping was shown. Interestingly, the detrimental influence of belonging to the first LTL quintile (i.e., having the shortest telomere lengths) on physical function remained evident (OR: 1.5, 95% CI: 1.1 – 2.1; *p* < 0.05) after combining all remaining quintiles of the study population as the reference group. Notably, there was no significant difference in handgrip strength between those having the shortest LTL compared to those with the longest LTL.

**Table 2:** Characteristics of the sub-sample population with the shortest (Q1) and the longest (Q5) LTL.

	Q1 LTL	Q5 LTL
	***n* = 240**	***n* = 233**
Having physical limitation^a^, %	38.3	25.8^∗^
Handgrip strength, kg	31.4 ± 9.7	31.9 ± 9.6
Medication, % yes	79.2	73.8
Former-current smokers, % yes	47.5	48.1
Overweight^b^, %	67.9	61.4
Study center,		
% France	16.3	15.9
% Italy	15.4	26.2
% Poland	32.5	7.7
% Netherlands	10.0	29.2
% United Kingdom	25.8	21.0


*LTL, Leucocyte Telomere Length. ^a^Based on composite construct. ^b^Based on BMI ≥ 25 kg/m^2^. Data expressed as mean ±*SD*, unless indicated. ^∗^*p* < 0.05 vs. Q1 LTL.*

## Discussion

This study examined links between LTL and physical functioning in a large European population of elderly men and women across diverse geographical locations. Collectively, our data highlight the existence of links between LTL and physical functioning, whereby individuals with the shortest LTL were more likely to experience functional limitations. This salient finding proposes that the detrimental effects of telomere length on age-related physical functioning do not follow a linear relationship but, instead are attributed to the occurrence of short LTL. The fact that this finding was evident across sex, age, geographical location, and overweight status highlights short LTL as an independent risk factor accounting for functional decline in aging European populations.

A novel finding in this study was that the influence of LTL on functional limitation was driven by the detrimental effect of having short telomeres, rather than reflecting a linear dose-response relationship. Previous investigations into the links between LTL and health outcomes have commonly reported weak to modest linear associations (Harris et al., 2006, 2012, 2016; Risques et al., 2010; Bendix et al., 2011; Lee et al., 2013; Woo et al., 2014; Sillanpää et al., 2016). The existence of a large inter-individual variability in LTL across study samples, together with a hypothesized non-linear relationship between LTL and function, may mask the true impact of having short telomeres on the development of age-related diseases (Scheinberg et al., 2010; Cui et al., 2012; Ye et al., 2013). Therefore, an analysis based on group comparisons of contrasting telomere lengths (the shortest vs. the longest telomeres) may enhance the ability of depicting the nature of telomere length influence on health outcomes. The higher likelihood of having functional limitations in elderly adults with the shortest LTL, compared to all other combined quintiles of LTL supports the notion of a threshold effect of telomere attrition, where short telomeres trigger replicative senescence (Allsopp et al., 1992) leading to cellular dysfunction in tissues and organs determining functional status. The existence of a non-linear relationship between LTL and physical functioning is further supported by the fact that our results from linear modeling were inconsistant, while those with the shortest LTL were more likely to have physical limitations compared to those with the longest. Our findings are in line with data indicating the increased risk of developing diabetes in subjects having short telomeres (lowest quartile) compared to those with longer LTL (Shen et al., 2012).

Another novel finding in the present study was that links between short LTL and physical functioning were observed despite the demonstrated sample variation in LTL. Indeed, our data reveal significant differences in LTL for elderly adults across European countries, in line with data reported in young adults across diverse European locations (Eisenberg et al., 2011). Geographical variations in LTL are poorly understood and may be attributed to both genetic and environmental factors. As our outcomes are based on participants from five geographically distinct European regions, covering different genetic and environmental backgrounds, the detrimental effects of short LTL on physical functioning may be generalized to broader elderly populations.

Interestingly, while short LTL is associated with the higher likelihood of having functional limitation, a corresponding link between LTL and handgrip strength was not observed. Previous studies based on single markers of physical function have yielded controversial results (Harris et al., 2006, 2012, 2016; Lee et al., 2013; Woo et al., 2014; Loprinzi and Loenneke, 2016; Sillanpää et al., 2016). As the use of single physical functioning markers may not fully capture the inability to perform activities of daily living, our data support the use of a composite construct of self-reported limitations in activities of daily living as an instrument that is more able to accurately reflect physical functioning.

The present study has limitations that should be acknowledged. First, the cross-sectional design restricts inferences on causality. Secondly, LTL can be estimated using southern blot or Q-PCR methods. Although both methods may yield comparable data, southern blot is considered as the gold standard (Kimura et al., 2010). Of note, the CV of our Q-PCR-based assessment of LTL was under 5%. Thirdly, even though physical function was assessed using objective and self-report methods, it may not fully cover all aspects of physical function. Finally, although several important variables were accounted for when exploring links between LTL and physical functioning, we cannot rule out the influence of other factors not addressed in the analysis.

## Conclusion

This study shows that elderly adults with shorter LTL are more likely to have functional limitations compared to those with longer LTL. The detrimental effects of telomere length on age-related physical functioning are not explained by a linear relationship, but attributed to the occurrence of short LTL. This finding was evident across diverse geographical locations, highlighting short LTL as an independent risk factor accounting for functional decline in elderly European populations.

## Author Contributions

All authors substantially contributed to the conception and design of the work, acquisition, analysis, and interpretation of the data. All authors drafted, critically reviewed, and approved the final version to be published.

## Conflict of Interest Statement

The authors declare that the research was conducted in the absence of any commercial or financial relationships that could be construed as a potential conflict of interest.

## Footnotes

1 www.nu-age.eu
